# Severe Iliopsoas Muscle Endometriosis Managed by a Multidisciplinary Team

**DOI:** 10.1097/og9.0000000000000064

**Published:** 2025-02-20

**Authors:** Harley Bray, Nicole Delaloye, Iain D. C. Kirkpatrick, Patricia Baker, Farhana Shariff, Devon Evans

**Affiliations:** Department of Obstetrics, Gynecology and Reproductive Sciences, the Department of Diagnostic Radiology, the Department of Pathology, and the Division of General Surgery, Department of Surgery, University of Manitoba, Winnipeg, Manitoba, Canada.

## Abstract

**BACKGROUND:**

There is limited published guidance on management of iliopsoas muscle endometriosis, especially when muscle is extensively infiltrated.

**CASE:**

A 38-year-old patient presented with left leg neuropathy, atrophy, edema, and catamenial bruising. Magnetic resonance imaging identified a poorly defined multiseptated cystic mass in the iliacus and inferior psoas muscle consistent with an endometrioma. After failed medical management, a multidisciplinary team was assembled for operative intervention. Excision was incomplete because the mass replaced most of the psoas muscle and obscured the femoral nerve. Postoperatively, pathology confirmed endometriosis and symptoms improved.

**CONCLUSION:**

In cases of iliopsoas endometriosis, surgical judgment should balance the dogmatic goal of complete excision with the ethical principle of nonmaleficence. Drainage and partial excision may allow for preserved muscle function and avoidance of catastrophic neurovascular injury.

## Abstract 2

A diagnosis of iliopsoas endometriosis should be considered in patients presenting with unilateral catamenial leg symptoms; this rare presentation requires multidisciplinary medical and surgical care.

**Teaching Points**

- Gynecologists and primary care clinicians should consider a diagnosis of iliopsoas endometriosis in reproductive-aged patients who present with unilateral femoral neuropathy, especially when cyclic exacerbations are reported.
- The femoral nerve runs between the iliacus and psoas muscle; care must be taken to medialize the iliac vessels and respect the femoral nerve when addressing an iliopsoas endometrioma.
- Complete excision of an iliopsoas endometrioma may be impractical or impossible due to limited or nonexistent surgical planes, the intimate relationships with the femoral nerve and iliac vessels, and the desire to optimize residual skeletal muscle function. Instead, incision, drainage, partial excision, or fulguration may be the best strategy.

Endometriosis is a chronic inflammatory condition estimated to affect 190 million people worldwide, with population-specific prevalence estimates ranging from 0.2% to 71.4% depending on geographical and clinical context of evaluation.^1,2^ The disease, characterized by extrauterine deposits of endometrial glands and stroma, most commonly affects reproductive-aged individuals. Involvement of genitourinary, gastrointestinal, and respiratory structures have been well described in the literature,^3–5^ but musculoskeletal involvement outside the abdominal wall is relatively rare.^6^ Iliopsoas endometriosis cases reported in the literature have been either medically managed,^7^ biopsied but not resected,^8^ or surgically resected, but none report extensive infiltration or replacement of the psoas muscle.^9^

Complete surgical excision of endometriosis is considered the standard of care in the setting of failed medical management.^10–12^ However, this may not be possible, particularly when anatomical planes are obliterated between major nerves and vessels or skeletal muscle is replaced by endometriosis. This report describes a unique presentation of neurologic, lymphatic, vascular, and musculoskeletal symptoms secondary to a left iliopsoas endometrioma essentially replacing the bulk of the psoas muscle. This case exemplifies the importance of multidisciplinary care and the balanced goals of complete excision and prevention of surgical harm.

## CASE

A 38-year-old patient, gravida 3 para 3, was referred to a minimally invasive gynecologic surgeon with gait disturbance and left leg pain on hip flexion, muscular atrophy, swelling, and catamenial groin bruising but normal bowel and bladder function. The patient’s medical history included three prior midline lower segment cesarean deliveries, a bicornuate-appearing uterus, and remote laparoscopy confirming endometriosis.

Initial examination findings included antalgic gait, left hip flexor weakness, and left-sided hypoesthesia in the distribution of the femoral, genitofemoral, and lateral cutaneous nerves. Dermatologic signs of vascular insufficiency over the left medial malleolus (Appendix 1, available online at http://links.lww.com/AOG/E5) and unilateral leg swelling were documented; deep vein thrombosis was ruled out by ultrasonography.

An initial computed tomography scan demonstrated left psoas muscular atrophy with a low-density collection along the anterior iliopsoas muscle measuring 3.0×4.1 cm. Subsequent magnetic resonance imaging (MRI) further characterized a multiseptated cystic mass in the anterior aspect of the left iliopsoas muscle (Appendix 2, available online at http://links.lww.com/AOG/E5).

This mass was consistent with an endometrioma measuring 3.2×4.4×7.6 cm. The lesion extended along the left external iliac vessels, with luminal narrowing. The previously known bicornuate uterus was identified, but there were no other deposits of deep endometriosis. The course of the femoral nerve was obscured by the mass and associated muscular invasion. The case was discussed with a multidisciplinary team composed of a minimally invasive gynecologic surgeon, a gynecologic radiologist, and a surgical oncologist. Based on symptoms, prior surgical diagnosis of endometriosis, and imaging findings, a diagnosis of left iliopsoas endometrioma was suspected.

The patient received 9 months of ovulatory suppression with leuprolide acetate with add-back therapy. Before their third injection, a follow-up MRI demonstrated a slight reduction in mass dimensions (3.2×4.4×7.6 cm to 2.9×4.0×5.4 cm). Despite regression in size, there was limited symptom improvement. The patient elected for aggressive surgical management including hysterectomy due to comorbid pelvic symptoms and family completion.

The team planned to start with laparoscopy for resection of any subtle endometriosis, hysterectomy, and potential management of the iliopsoas mass. We planned for possible conversion to laparotomy depending on intraoperative findings and accessibility of the mass. Careful inspection of the pelvis surprisingly revealed no evidence of superficial or deep endometriotic implants. There was adhesive disease from the prior cesarean deliveries between the anterior abdominal wall, bladder, and uterus. The upper abdomen and appendix were normal. The patient underwent cystoscopy with insertion and removal of bilateral lighted ureteric stents, total laparoscopic hysterectomy, bilateral salpingectomy, lysis of adhesions, and left oophorectomy sparing the right ovary. Although the uterus was bicornuate-appearing on imaging, surgical findings were consistent with a unicornuate right uterus and left noncommunicating horn (Appendix 3, available online at http://links.lww.com/AOG/E5).

On laparoscopic assessment of the mass with the surgical oncologist, it was difficult to distinguish between iliac vessels and cystic mass. For improved tactile feedback and visualization, we converted to an infraumbilical midline laparotomy for the remainder of the case. The ureter and iliac vessels were identified and medially reflected after extensive arterial dissection. The mass was interdigitating with iliopsoas muscle fibers such that the femoral nerve was unidentifiable. The mass was incised and drained, and a subtotal resection was performed to prevent closure and recurrence (Appendix 4, available online at http://links.lww.com/AOG/E5). Fulguration of the endometrioma bed resulted in active contraction of femoral nerve–supplied muscles. A vascular surgeon was consulted intra-operatively to confirm that a complete neurolysis could not be performed safely due to the density of adhesions and fibrosis. Trabeculated tissue consistent with fibrotic, endometriosis-infiltrated skeletal muscle was identified and fulgurated at the base of the unresectable capsule, and the roof of the lesion was excised to prevent reaccumulation and obtain pathologic assessment (Appendix 5, available online at http://links.lww.com/AOG/E5). Histopathologic evaluation of the left retroperitoneal specimen confirmed fibrofatty tissue with endometriosis and no malignancy (Appendix 6, available online at http://links.lww.com/AOG/E5).

The patient's postoperative course was uneventful. Continuous ovulatory suppression was maintained with drospirenone. By 6 weeks postoperatively, there was nearly complete resolution of preoperative gait disturbance and pelvic pain. However, there was some residual pain with hip flexion and ongoing paresthesia. A referral was made for physical therapy, and amitriptyline was continued for neuromodulation. At 5 months postoperatively, there was further improvement in neurologic symptoms, including power with hip flexion, thigh sensation, and complete normalization of gait. The dermatologic manifestations of vascular insufficiency persisted, but the edema resolved. Four months postoperatively, an MRI showed a small residual endometriotic implant along the left iliopsoas muscle measuring 1.6×0.6 cm (Appendix 7, available online at http://links.lww.com/AOG/E5).

## DISCUSSION

A recent systematic review of endometriosis of skeletal muscle identified only five cases of psoas or iliopsoas endometriosis.^6^ On our review of the literature, we identified one additional case that appeared to include the renal hilum and extended to the iliopsoas muscle that was managed with open excision.^13^ We also identified one case of a less invasive retro-psoas endometrioma successfully managed medically; this is the only report of adequate medical management absent surgical intervention.^7^

In published cases of iliopsoas endometriosis, patients have presented with back, hip, or leg pain, with or without lower extremity neuropathy, and imaging findings such as hydronephrosis or a heterogenous, fibrous or cystic lesion displacing the psoas muscle.^7–9,13–17^ In two of the surgically managed cases, the endometriomas were mistaken for psoas muscle abscesses before pathologic assessment.^9,17^ There are only two reported cases of laparoscopic resection of iliopsoas endometriosis, one of which was a multistage, multidisciplinary procedure^16^; the other describes less deeply invasive endometriosis extending 12 cm along the surface of the psoas muscle.^9^ The lesion described was confined to the surface of the psoas muscle, allowing for discernment of planes. The other cases either only biopsied the lesion,^8^ drained it with computed tomography guidance,^15^ or performed open excision, which may not have been complete.^13,17^ We present the eighth reported case, which is a unique presentation of endometriosis essentially replacing the bulk of the left psoas muscle and encasing the femoral nerve.

Endometriosis-associated neuropathy can generate chronic pain and nerve damage from fibrosis, inflammation, and bleeding.^18^ Muscular denervation from endometriosis infiltration can lead to muscular atrophy, as seen in this case.^19^ Early diagnosis of neural involvement in endometriosis is imperative, because timely treatment may prevent irreversible nerve damage and chronic neuropathies.^20^ Vascular compromise from endometriotic lesions is likely from mechanical compression resulting in luminal narrowing.^21^ Hormonal suppression should be initiated early to optimize symptoms and determine the need for surgical intervention. If malignancy cannot be excluded or mechanical symptoms persist with manifestations of nerve entrapment, compression, or irritation or vascular or lymphatic obstruction, one should consider assembling a multidisciplinary surgical team with expertise in all anatomic distributions involved.

Preoperative hormonal suppression may shrink implants and reduce inflammation to improve surgical outcomes.^6^ However, as was presented here, even with optimal preoperative suppression and assembly of a multidisciplinary team, complete resection may not always be the best approach. In this case, an endometrioma almost entirely replaced the left iliopsoas muscle such that complete resection was not feasible without high likelihood of damage to the femoral nerve. Medical treatment, coupled with drainage and partial resection, afforded this patient markedly improved gait and pelvic pain and slowly improving paresthesia.

In conclusion, it is important to consider a diagnosis of iliopsoas endometriosis when patients present with musculoskeletal, neurologic, or lymphatic leg symptoms of unclear etiology, especially when cyclic exacerbations are reported. In cases requiring surgery, deep endometriosis may exist even without evidence of peritoneal implants. Meticulous dissection is required to avoid damage to the femoral nerve and iliac vessels, which can be medialized to expose the iliopsoas complex. The dogmatic recommendation of complete excision may be impossible or impractical due to limited or nonexistent surgical planes, the intimate relationships with the femoral nerve and iliac vessels, or the desire to optimize residual skeletal muscle function. Instead, drainage, partial excision, fulguration, or a combination of these may sometimes be the best strategy, augmented by postoperative therapies.
